# Successful Use of Renal Replacement Therapy for Refractory Hypokalemia in a Diabetic Ketoacidosis Patient

**DOI:** 10.1155/2019/6130694

**Published:** 2019-08-14

**Authors:** Zainab Syed, Thomas Kimball, Mourad Ismail

**Affiliations:** ^1^Department of Medicine, Saint Joseph's University Medical Center, 703 Main St, Paterson, NJ, USA; ^2^New York Medical College, Valhalla, NY, USA

## Abstract

A 39-year-old African-American female presented to the emergency department with a seven-day history of right upper quadrant abdominal pain accompanied by nausea, vomiting, and diarrhea. She was noted to be alert and following commands, but tachypneic with Kussmaul respirations; and initial laboratory testing supported a diagnosis of diabetic ketoacidosis (DKA) and hypokalemia. To avoid hypokalemia-induced arrhythmias, insulin administration was withheld until a serum potassium (K) level of 3.3 mEq/L could be achieved. Efforts to increase the patient's potassium level via intravenous repletion were ineffectual; hence, an attempt was made at more aggressive potassium repletion via hemodialysis using a 4 mEq/L K dialysate bath. The patient was started on Aldactone and continuous veno-venous hemodialysis (CVVH) with ongoing low-dose insulin infusion. This regimen was continued over 24 h resulting in normalization of the patient's potassium levels, resolution of acidosis, and improvement in mental status. Upon resolution of her acidemia, the patient was transitioned from insulin infusion to treatment with a subcutaneous insulin aspart and insulin detemir, and did not experience further hypokalemia. Considering our success, we propose CVVH as a tool for potassium repletion when aggressive intravenous (IV) repletion has failed.

## 1. Introduction

Hospitalizations for diabetic ketoacidosis (DKA) have soared in incidence over the recent years, increasing 54.9% from 19.5 to 30.2 hospitalizations per 1,000 people with diabetes in 2009–2014 alone. According to the CDC, DKA is responsible for more than 500,000 hospital days per year incurring an estimated annual expense of 2.4 billion dollars. Despite increasing incidence, the mortality rate for DKA has declined significantly [1]. Its successful management requires not only addressing the patient's underlying acidosis but also carefully managing co-existing electrolyte abnormalities. Patients presenting with diabetic ketoacidosis are usually hyperkalemic secondary to their acidemia but exhibit low levels of total body potassium, necessitating potassium repletion concurrent with insulin therapy. We present a case of a newly diagnosed diabetic patient who presented with DKA confounded by severe total body potassium depletion, manifesting as sustained hypokalemia refractory to potassium repletion. Her serum potassium level was finally corrected using renal replacement therapy, allowing insulin to be administered and providing a complete recovery for the patient.

## 2. Case Presentation

A 39-year-old African-American female presented to the emergency department with a seven-day history of right upper quadrant abdominal pain accompanied by nausea, vomiting, and diarrhea. The patient was seen in the emergency department three days earlier for an asthma exacerbation, and was discharged with prescriptions for albuterol and a tapered prednisone regimen. On exam at presentation, she was noted to be alert, following commands, and neurologically intact with tachypnea and Kussmaul respirations. A more detailed history was unavailable. Her blood pressure was 137/76 mmHg, her heart rate was 98 beats per minute, and her respiratory rate 26 breaths per minute.

Initial laboratory testing revealed a blood glucose level of 676 mg/dL, a serum potassium level of 3.0 mEq/L, a corrected serum sodium level of 121 mEq/L, a serum chloride level of 84 mEq/L, and a serum magnesium level of 2.9 mg/dL with an anion gap of 30. In addition, renal function tests showed acute kidney injury with a blood urea nitrogen (BUN) of 32 mg/dL and a creatinine of 1.55 mg/dL, both elevated from her baseline BUN of 10 mg/dL and creatinine of 0.69 mg/dL from 18 months prior to this hospitalization. Arterial blood gas analysis showed a blood pH of 7.14 and a serum *β*-hydroxybutyrate level of 99 mg/dL, confirming the diagnosis of DKA.

The patient was admitted to the Medical Intensive Care Unit (MICU). To avoid hypokalemia-induced arrhythmias, insulin administration was withheld until a serum potassium level of greater than 3.3 mEq/L could be achieved. Potassium sparing diuretics were also avoided due to the patient's previously mentioned low serum sodium level. While the patient's low serum sodium eventually corrected after administration of 250 mEq of sodium bicarbonate, initial repletion with 50 mEq of IV K resulted in only a slight increase from 3.0 to 3.1 mEq/L. Ongoing efforts to further increase the patient's potassium level over the next 18 h were unsuccessful. Despite receiving 340 mEq of IV K, her serum level decreased to 2.9 mEq/L, further delaying the administration of insulin therapy. At this juncture, the patient's clinical status began to deteriorate. She became lethargic with a nonfocal neurological exam. Her heart rate increased to 137, and her electrocardiogram began to show new T-wave inversions in the inferior and lateral leads with increasingly prominent U-waves. Her acidemia also worsened with arterial blood pH falling to 7.05.

In view of our patient's refractory hypokalemia and her rapidly deteriorating condition, a decision was made to start a more aggressive potassium repletion via hemodialysis. Following consultations with a nephrologist, the patient was dialyzed using a bath containing 4.0 mEq/L potassium and 40 mEq/L bicarbonate. Following dialysis, the patient's serum potassium level temporarily improved to 3.6 mg/L, her BUN and creatinine fell to 21 mg/dL and 0.87 mg/dL, respectively, and an insulin infusion was initiated with IV ongoing K replacement. However, repeat serum chemistry four hours later showed a drop in potassium level to 2.6 mEq/L. At this point, the patient was started on Aldactone and placed on continuous veno-venous hemodialysis (CVVH) using a 4.0 mEq/L dialysate with ongoing low-dose insulin infusion. Her potassium level increased and remained above 3.5 mEq/L. CVVH along with insulin therapy was continued over the next 24 h with rapid normalization of the patient's potassium levels, resolution of her acidosis, and improvement in her mental status. Renal function improved approaching baseline with a BUN of 16 mg/dL and creatinine of 0.88 mg/dL observed 10 h after the initiation of CVVH therapy. Successful insulin therapy also ended our patient's osmotic diuresis and allowed for correction of her substantial dehydration with our patient body weight increasing from 88.4 kg on hospital admission to 95.4 kg on discharge from the MICU. A summary of the temporal progression of the patient's electrolyte values is referenced in Table 1.

Upon resolution of her anion gap metabolic acidosis, the patient was transitioned from an insulin infusion to treatment with a combination of subcutaneous insulin Aspart and insulin Detemir, and did not experience further hypokalemia. She was transferred out of the MICU and continued to receive insulin therapy. Her serum hemoglobin A1C was found to be 12%, and our patient was discharged with a diagnosis of insulin requiring diabetes mellitus with plans for further outpatient endocrinology workup.

## 3. Discussion

This patient's presentation was both unique and challenging. Hypokalemia is infrequently seen at presentation in DKA, with recent studies estimating that it occurs in fewer than 6% of DKA patients arriving at emergency departments [2, 6]. In this case, our patient's use of an albuterol inhaler for asthma exacerbation may have contributed to her hypokalemia by shifting potassium intracellularly, yet this does not explain its refractory presentation.

We believe that our patient's refractory hypokalemia was a reflection of a profound total body potassium deficit which we were unable to correct rapidly enough using simple potassium infusion. Her initial complaint of seven days of vomiting and diarrhea suggests that she had suffered prolonged gastrointestinal losses of potassium. This would be congruent with our patient's low serum levels of multiple other electrolytes on presentation.

We initially considered other differential diagnoses while attempting to treat this patient. An endocrine cause seemed unlikely. Our patient did initially demonstrate an elevated blood cortisol level, but her physical exam and hyponatremia were not consistent with Cushing's syndrome. Similarly, an intrinsic renal cause for this patient's hypokalemia was improbable. The presence of a metabolic acidosis and not a metabolic alkalosis in this patient ruled out both Barter's and Liddle's syndromes, while her low serum chloride level was incongruent with the hyperchloremia that would be expected in a distal renal tubular acidosis. Throughout her first few days in the hospital, multiple serum magnesium levels were tested, producing results that were high-normal or elevated. For this reason, we did not suspect hypomagnesemia to be a causal factor to our patient's refractory hypokalemia, and did not initially administer IV magnesium.

Our hospital's internal protocols for potassium replacement and insulin administration in DKA patients specify the addition of 20 mEq K for serum potassium levels from 4.5 mEq/L to 5.5 mEq/L and 40 mEq K for levels below 4.4 mEq/L per liter of IV fluids infused. Insulin is withheld in cases where serum potassium is less than or equal to 3.3 mEq/L. Potassium infusion rates are limited to 20 mEq/h when given via a central intravenous catheter. We initially attempted to follow these protocols but our patient's profound potassium deficit led to a severe delay in the correction of her hypokalemia. Despite 18 h of treatment with the maximum permissible dose of intravenous potassium, her serum potassium level remained unchanged from that seen at her admission.

Although there are case reports of hypokalemia in DKA patients that required aggressive potassium replacement, all but one responded to initial potassium repletion [2, 3, 4, 6]. Faced with a unique case of a DKA patient with hypokalemia unresponsive to intravenous replacement, combined with active clinical deterioration, we opted to use renal replacement therapy as a salvage method to correct the potassium deficit, thereby allowing for the safe administration of insulin. Although this is an aggressive treatment approach, we believe that it was warranted in this case given the clinical scenario. After a review of the literature, we believe that this is the first reported use of CVVH for this purpose.

Using CVVH in the setting of DKA presents several challenges. Studies have shown that increased bicarbonate levels in dialysate fluid may induce further hyperglycemia [5]. This combined with the increased clearance of insulin with hemofiltration would mandate the administration of higher insulin doses. However, care must be taken to not administer excessive insulin, so as to avoid inducing further hypokalemia or rapid osmotic shifts. In this case we chose to use a dialysate solution with bicarbonate content of 40 mEq/L due to the patient's profound metabolic acidosis, while using a lower dose insulin infusion—4 U/h for this 88.6 kg patient. During CVVH, the patient's serum osmolality remained stable within a range of 16 mmol/l. By successfully balancing the effects of CVVH and insulin on glucose and electrolyte concentrations, we were able to successfully treat our patient while avoiding complications such as arrhythmias and cerebral edema.

## 4. Conclusion

Here we present a case of severe refractory hypokalemia in the setting of DKA, which ultimately improved with the use of CVVH, allowing the patient to recover fully. Based on our experience, we propose that when aggressive replacement of potassium is warranted and intravenous repletion remains inadequate, CVVH might be a valuable salvage therapy to expedite potassium repletion.

## Figures and Tables

**Table 1:** Timeline of selected laboratory values and treatments in our patient.

Hours post admission	Corrected sodium (mEq/L)	Potassium (mEq/L)	BUN (mg/dL)	Creatinine (mg/dL)	Magnesium (mg/dL)	Arterial pH
0	121	3.0	32	1.55	2.9	7.14
*Administration of IV 50 mEq KCl, 1000 ml NaCl*						
5	123	3.1	28	1.11	2.5	7.09
*Administration of IV 290 mEq KCl, 250 mEq NaHCO_3_*						
18	151	2.9	29	1.11	2.6	7.05
*Initiation of hemodialysis with 4 mEq/L K-dialysis bath, 220 ml of positive fluid balance*						
25	147	3.6	21	0.87	2.4	7.47
*Administration of IV insulin 4 U/h, 40 mEq KCl, 30 mmol K-Phos*						
29	146	2.6	18	0.86	2.0	7.43
*Initiation of CVVH, 1,550 ml of positive fluid balance*						
32	141	3.4	21	1.05	1.9	7.39
40	145	4.1	16	0.88	2.1	7.39

## References

[1] Gosmanov A. R., Gosmanova E. O., Kitabchi A. E., Feingold K. R., Anawalt B., Boyce A. (2000). Hyperglycemic crises: diabetic ketoacidosis (DKA), and hyperglycemic hyperosmolar state (HHS) [Updated 2018 May 17]. *Endotext*.

[2] Jang T. B., Chauhan V., Morchi R., Najand H., Naunheim R., Kaji A. H. (2015). Hypokalemia in diabetic ketoacidosis is less common than previously reported. *Internal and Emergency Medicine*.

[3] Davis S. M., Maddux A. B., Alonso G. T., Okada C. R., Mourani P. M., Maahs D. M. (2016). Profound hypokalemia associated with severe diabetic ketoacidosis. *Pediatric Diabetes*.

[4] Murthy K., Harrington J. T., Siegel R. D. (2005). Profound hypokalemia in diabetic ketoacidosis: a therapeutic challenge. *Endocrine Practice*.

[5] Takahashi A., Kubota T., Shibahara N. (2004). The mechanism of hypoglycemia caused by hemodialysis. *Clinical Nephrology*.

[6] Arora S., Cheng D., Wyler B., Menchine M. (2012). Prevalence of hypokalemia in ED patients with diabetic ketoacidosis. *The American Journal of Emergency Medicine*.

